# Awareness About Glaucoma Management Among Physicians in Riyadh: A Multicenter Cross-Sectional Study

**DOI:** 10.7759/cureus.8450

**Published:** 2020-06-05

**Authors:** Manal Alwazae, Atheer Almutairi, Atheer Alhumud, Alhanouf Alhumidan, Areej A Alqahtani, Sumera Nisar

**Affiliations:** 1 Ophthalmology, College of Medicine, Princess Nourah Bint Abdulrahman University (PNU), Riyadh, SAU; 2 Ophthalmology, Princess Nourah Bint Abdulrahman University (PNU), Riyadh, SAU; 3 Pediatrics, College of Medicine, Princess Nourah Bint Abdulrahman University (PNU), Riyadh, SAU; 4 Ophthalmology, Retina Consultant, Jeddah, SAU

**Keywords:** glaucoma practice, primary health care, blindness prevention

## Abstract

Purpose

Glaucoma is an irreversible chronic disease that damages the optic nerve. Knowledge and skills related to glaucoma are extremely important for frontline physicians. This study aimed to determine the knowledge and management as well as examination and referral practices related to glaucoma among physicians at primary care centers and secondary hospitals in Riyadh, Saudi Arabia.

Method

This was a cross-sectional study of 126 physicians, including general practitioners as well as emergency, internal, and family physicians from three hospitals and five medical centers in Riyadh. A validated self-administered questionnaire was used for data collection. It was divided into six categories: sociodemographic data and practice setting; and glaucoma assessment-questions regarding risk factors, knowledge, examination, management, and referral practices.

Results

Of the 126 participants, 32.8% were family physicians. Surprisingly, the overall knowledge score for glaucoma was suboptimal (34.2%). While half of the doctors were aware of the medications used in glaucoma, 88.7% considered themselves unqualified to manage glaucoma. Although 93.7% agreed that increased ocular pressure requires urgent referral to an ophthalmologist, only 33.3% stated they were comfortable using tonometry.

Conclusion

The majority of physicians (65.8%) showed a poor level of knowledge regarding glaucoma, which was reflected in their referral practices (66.9%). Therefore, promoting increased glaucoma awareness along with improved examination skills and referral practice among frontline physicians is essential to prevent this avoidable cause of blindness.

## Introduction

Glaucoma is a condition that causes damage to the optic nerve, and it worsens over time if left untreated. It is often linked to a build-up of pressure inside the eye. Essentially, it is a chronic, irreversible disease that ultimately leads to optic neuropathy [1]. It is also a leading cause of blindness worldwide [2]. Globally, the prevalence of glaucoma is 3.54% in people above 40 years in age, which was estimated to be 64.3 million people in 2013, increasing to 76 million in 2020 [3]. In Saudi Arabia, the prevalence of blindness is 1.5%, and glaucoma is responsible for 3% of all blindness in patients over 40 years old [4,5]. Therefore, glaucoma is considered to be one of the most common causes of blindness in Saudi Arabia.

A previous study has suggested that factors contributing to the development of glaucoma are age over 40 years, a family history of glaucoma, African race, and chronic diseases (e.g., diabetes mellitus, hypertension, and migraine) [6]. Due to its silent progressive nature, early detection through a full ocular examination is needed for at-risk patients. The full ocular examination includes measuring intraocular pressure (IOP), the visual field, and the cup-to-disc ratio. Thus, screening should assess all those aspects. One study failed to find a correlation between the development of glaucoma and high IOP levels alone [7]. However, the decision to seek medical care and early diagnosis may be altered by the absence of symptoms, which contributes to the fact that around half of patients are either undiagnosed or not treated in a timely manner [8,9].

Interestingly, 50% of diagnosed glaucoma cases present late and are revealed to have moderate to severe disease at initial diagnosis [8,10,11]. Moreover, managing patients with glaucoma can be challenging as it requires long-term follow-ups and adherence to the recommended treatment [12]. Therefore, the knowledge and skills of physicians in primary care centers and secondary hospitals regarding glaucoma are extremely important, as they are the frontline screeners in the healthcare system in Saudi Arabia; besides, they are the physicians closest to the patients [13]. In fact, referrals from family physicians account for 7% of the total glaucoma case referrals [8]. Hence, increasing physician awareness of glaucoma care practice could contribute greatly to preventing this leading cause of irreversible blindness [14].

To date, there is limited data in Saudi Arabia regarding the role of physicians at primary care centers and family physicians at secondary hospitals in glaucoma care. Hence, this study aimed to determine the knowledge and referral practices of physicians related to glaucoma in Riyadh, Saudi Arabia.

## Materials and methods

Design, setting, and participants

This cross-sectional study was conducted in three secondary hospitals and five primary care centers in Riyadh city, the capital of Saudi Arabia, from December 2018 to May 2019. We enrolled a total of 126 physicians, including general practitioners, family physicians, internists, and emergency medicine physicians. Family medicine physicians were enrolled from secondary hospitals and primary care centers. Of note, physicians at primary care centers in Saudi Arabia could be from diverse backgrounds as long as they have a diploma in family medicine. Therefore, internists, emergency medicine physicians, and general practitioners were recruited only from primary care centers, and they had the required credentials to work in primary care centers.

We calculated our sample size based on the expected level of physician awareness about glaucoma using the free G*Power online software (release 3.1.9.2). Considering a level of glaucoma awareness of 70% ± 13% and a 95% confidence interval (CI) at a level of significance (α = 0.05) and a power of 80% (β = 0.2), the estimated minimum sample size was 114, which was increased to 126 to compensate for any incomplete data.

Data collection

A validated self-administered questionnaire was adapted from a Canadian study and used for data collection [8]. The questionnaire included six main sections: sociodemographic and practice setting, glaucoma risk factors, knowledge, examination, management, and referral. Sociodemographic data and practice setting included age, gender, position (resident, fellow, consultant), specialty, and frequency of seeing glaucoma patients. The questions assessed glaucoma in terms of risk factors (seven questions), knowledge (six questions), examination (four questions), management (six questions), and referral practices (seven questions). The responses were rated on a five-point Likert scale, ranging from 1 (strongly disagree) to 5 (strongly agree).

The total knowledge score was computed, and the variable was then transformed into a dichotomous measure. After consulting an expert in public health and epidemiology (Prof. Hala Elmorshedy), we defined adequate knowledge among participants as the ability to answer more than three questions (≥70%); participants were deemed to have inadequate knowledge if they could answer only three or fewer questions (≤50%). The same rule applied to all glaucoma assessment questions. Notably, all glaucoma assessment questions were based on the Canadian Ophthalmological Society evidence-based clinical practice guidelines for the management of glaucoma in adults [15].

Data analysis

The statistical analysis was conducted using SPSS Statistics version 23 (IBM Corp., Armonk, NY). The quantitative variables were described in means and standard deviations (SDs), and the qualitative variables were described in proportions. The association of glaucoma knowledge and referral practice was assessed with a chi-squared test, and p-values of <0.05 were considered statistically significant. We divided the Likert scale into two categories: agree (strongly agree, agree) and disagree (neutral, disagree, and strongly disagree).

Ethical consideration

The Institutional Review Board (IRB) approval number 18-0239 was obtained from the IRB Committee of Princess Nourah Bint Abdulrahman University. All participants provided verbal consent prior to enrollment.

## Results

Table 1 presents the sociodemographic data and practice settings. A total of 126 physicians were involved in this study. They had a mean age of 37 ± 8 years; 58% were male and 32.8% were family physicians, and 44.3% were residents in training. A minority of the physicians (36.6%) reported that they saw patients with a diagnosis of glaucoma less than annually. The majority of physicians (77.9%) reported that ophthalmologists provide the primary management for patients with glaucoma.

**Table 1:** Demographic data and practice setting SD: standard deviation

Variables	Values
Age in years, mean ± SD	37 ± 8
Male, n (%)	76 (58%)
Female, n (%)	45 (34.4%)
Position:	
Resident, n (%)	58 (44.3%)
Fellow, n (%)	15 (11.5%)
Consultant, n (%)	48 (36.6%)
Specialty:	
General practitioner, n (%)	31 (23.7%)
Family physicians, n (%)	43 (32.8%)
Emergency medicine, n (%)	16 (12.2%)
Internal medicine, n (%)	36 (27.5%)
How often do you see glaucoma patients?	
Daily, n (%)	2 (1.5%)
Weekly, n (%)	7 (5.3%)
Monthly, n (%)	21 (16%)
Semiannually, n (%)	36 (27.5%)
Annually, n (%)	12 (9.2%)
Less frequently, n (%)	48 (36.6%)
Who provides primary management to your patients who are diagnosed with glaucoma?	
Primary care physicians, n (%)	5 (3.8%)
Ophthalmologist, n (%)	102 (77.9%)
Optometrist, n (%)	4 (3.1%)
Ophthalmologist or optometrist, n (%)	10 (7.6%)
Not sure, but not the primary care physicians, n (%)	5 (3.8%)

The majority of the physicians correctly agreed that the following items are risk factors for developing glaucoma: advancing age (85.6%), steroid use (79.4%), and family history (77.6%). Regarding general knowledge about glaucoma, 93.7% of the physicians agreed that blindness could be prevented if glaucoma is detected and treated early, and 61.1% recognized that early glaucoma does not present with central visual loss. However, most physicians did not consider themselves qualified to use tonometry (66.7%), and only 48.4% were comfortable examining the optic nerve with a direct ophthalmoscope. Considering different modalities of glaucoma treatment, 56.3% of the doctors were aware of the medications used in glaucoma treatment. However, only around half of them were aware of the side effects of the medications. Most physicians (93.7%) agreed that patients should be referred to an ophthalmologist if there was an increase in IOP. In comparison, only 71.2% agreed a referral was necessary if the patient’s cup-to-disc ratio was greater than 0.5 (Table 2).

**Table 2:** Glaucoma assessment questions

Questions	Agree, n (%)	Disagree, n (%)
Which of the following are risk factors for open-angle glaucoma? (Risk factors)		
Advancing age	107 (85.6%)	18 (14.4%)
Family history	97 (77.6%)	28 (22.4%)
African descent	53 (42.4%)	72 (57.6%)
Chronic corticosteroid use	100 (79.4%)	26 (20.6%)
High blood pressure	94 (75.2%)	31 (24.8%)
Male gender	64 (51.2%)	61 (48.8%)
Last completed eye examination more than 5 years ago	42 (33.6%)	83 (66.4%)
To what extent do you agree with each of the following statements? (Knowledge)		
Blindness can be prevented if glaucoma is detected and treated early	118 (93.7%)	8 (6.3%)
Early open-angle glaucoma is symptomatic	63 (50%)	63 (50%)
Early glaucoma involves central visual field loss	49 (38.9%)	77 (61.1%)
Visual field loss can be detected after minor damage to nerve fibers	58 (46%)	68 (54%)
I am aware of the pathophysiology of glaucoma	87 (69%)	39 (31%)
I am familiar with the Canadian Ophthalmological Society guidelines for the management of glaucoma	37 (29.4%)	89 (70.6%)
To what extent do you agree with each of the following statements? (Examination)		
I am comfortable performing tonometry	42 (33.3%)	84 (66.7%)
I am comfortable examining the optic nerve for glaucoma using the direct ophthalmoscope	61 (48.4%)	65 (51.6%)
I am comfortable assessing a visual field	90 (71.4%)	36 (28.6%)
I am comfortable checking for a relative afferent pupillary defect	79 (62.7%)	47 (37.3%)
To what extent do you agree with each of the following statements? (Management)		
I am comfortable managing general conjunctival and corneal diseases	73 (58.4%)	52 (41.6%)
I am comfortable managing optic nerve and retinal diseases	40 (31.7%)	86 (68.3%)
I am aware of the medications used in glaucoma	71 (56.3%)	55 (43.7%)
I am aware of the side effects of medications used in glaucoma	67 (53.6%)	58 (46.4%)
I am aware of the different lasers used in glaucoma	41 (32.5%)	85 (67.5%)
I am aware of the different operations used in glaucoma	47 (37.3%)	79 (62.7%)
To what extent do you agree with each of the following statements? (Referral)		
Increased eye pressure	118 (93.7%)	8 (6.3%)
Peripheral visual field loss only	107 (84.9%)	19 (15.1%)
Central visual field loss	109 (86.5%)	17 (13.5%)
Presence of risk factors for glaucoma	92 (74.2%)	32 (25.8%)
Cup-to-disc ratio greater than 0.5	89 (71.2%)	36 (28.8%)
Cup-to-disc ratio greater than 0.1 between the two eyes	75 (60%)	50 (40%)
Eye pain, nausea, and colored halos	109 (86.5%)	17 (13.5%)

Table 3 illustrates the participants’ overall glaucoma knowledge, examination skills, management, and referral practices scores. The overall knowledge score was poor for 65.8% of the physicians. The majority of physicians (65.1%) were not trained to perform ocular examinations, whereas 66% had good referral practice. Additionally, we did not find a significant association between glaucoma knowledge and referral practice (p=0.31). Moreover, we did not find a significant association between glaucoma knowledge and medical degree of participants (p=0.43) or their background (p=0.39).

**Table 3:** Overall scores

Overall scores	Good	Poor
Knowledge	34.2%	65.8%
Examination skills	34.9%	65.1%
Management	11.3%	88.7%
Referral practice	66.9%	33.1%

## Discussion

The physicians’ scores on glaucoma knowledge, examination skills, and management were found to be low (34.2%, 34.9%, and 11.3%, respectively). Furthermore, the majority had suboptimal referral practices (66.9%). Although almost half of the participants were residents in training, and we did not find a significant association between glaucoma knowledge and medical degree (p=0.43). Unfortunately, this could affect the physician’s role in the early detection of high-risk patients and timely referral to an ophthalmologist.

Looking specifically at the knowledge of glaucoma risk factors, most of the physicians agreed that family history, advancing age, and steroid use were strong risk factors for glaucoma development. Nevertheless, ophthalmologists expect primary care physicians to refer patients based mainly on risk factors rather than on examinations, which require special training and specific instruments [8]. Steroid use is considered a particularly important factor, and physicians at primary care centers should be aware of its role in the development of glaucoma. Physicians could acquire this information by merely performing histories and examinations and asking the patient about the use of any type of local or systemic steroid, including steroid injections for diabetic macular edema [16,17].

The general level of knowledge about glaucoma was poor among our respondents (34.2%), and almost half of them were unaware of the side effects of glaucoma medications. Similarly, 46% of primary care physicians in Rotshtein et al.’s study reported that they received inadequate knowledge about glaucoma during their training, and 54% were unfamiliar with the side effects of glaucoma medications [18]. Hence, the development of guidelines for glaucoma management targeting frontline physicians would help them to understand glaucoma medications and side effects so they can effectively counsel glaucoma patients.

Interestingly, the majority of the participants (93.7%) agreed that they should refer patients with high IOP. However, only 33.3% of them were comfortable using tonometry. Similarly, a Canadian study reported that 97% of family physicians would refer patients with high IOP, but only 30% were able to use tonometry [8]. Examination skills, including the use of tonometry and direct ophthalmoscopes, have been discussed thoroughly in the literature. Shuttleworth et al. found that only 56% of primary care practitioners were confident in performing direct ophthalmoscope examinations [19]. In Bell et al.’s study, only 9% of glaucoma referrals came from general practitioners, and those that did were based on histories alone [20]. This highlights the need to train physicians at primary care centers to perform ocular examinations to detect patients needing referral to an ophthalmologist based on high IOP.

The results of our study could provide stakeholders and program directors of training programs with information regarding areas that need to be improved in the curriculum, with an emphasis on increasing knowledge about glaucoma management and risk factors and providing ocular examination training to support better referral practices. Nevertheless, physicians at primary care centers play a crucial rule in blindness prevention by ensuring that high-risk patients are screened and by supporting patients’ optimal adherence to medical treatment [21-23].

Limitations

This study has some limitations. Our sample consisted solely of physicians and residents in training at primary care centers and secondary hospitals in Riyadh and the data cannot be generalized.

## Conclusions

This study revealed significant gaps in the knowledge about glaucoma among physicians at secondary hospitals and primary care centers in Riyadh, which was reflected in their referral practices. We believe that promoting increased glaucoma awareness and improved examination skills and referral practices among frontline physicians are essential to aid in the prevention of this leading cause of irreversible blindness.
